# Carbon dioxide digital subtraction enterography for route identification in post-Roux-en-Y biliary interventions

**DOI:** 10.1055/a-2523-2886

**Published:** 2025-02-11

**Authors:** Akihiro Maruyama, Makoto Kobayashi, Hirotaka Takeshima, Hiroshi Nakayabu, Hiroki Kato, Shintaro Tominaga, Motoyoshi Yano

**Affiliations:** 1 37036 Department of Gastroenterology, Yokkaichi Municipal Hospital, Yokkaichi, Japan

Endoscopic retrograde cholangiography (ERC) with balloon-equipped endoscopy is used for treating biliary diseases in patients with altered gastrointestinal anatomy. Carbon dioxide enterography is commonly used during ERC for route selection at the anastomotic branch, as it allows noninvasively visualizing intestinal pathways 1 2 . However, route identification accuracy may be compromised in cases with excessive intestinal gas, where overlapping shadows obscure the anatomy. Digital subtraction imaging (DSI) is a radiological technique widely used to enhance visualization. By subtracting a pre-contrast image from a post-contrast image, DSI allows for clear identification of vascular and anatomical structures while minimizing interference from surrounding tissues 3 . When applied to carbon dioxide enterography, this technique is termed carbon dioxide digital subtraction enterography. To acquire DSI sequences, we used the Ultimax-i DREX-U180 (Canon, Tokyo, Japan). In ERC, DSI can improve the effectiveness of carbon dioxide enterography, particularly in challenging cases involving excessive intestinal gas. This report highlights the successful use of carbon dioxide digital subtraction enterography in overcoming the limitations of traditional imaging and enabling better visualization.

A 77-year-old man with a history of total gastrectomy and Roux-en-Y reconstruction presented with abdominal pain. Drip infusion cholangiography-computed tomography revealed common bile duct stones ( Fig. 1 ). The patient was scheduled to undergo ERC with a single-balloon endoscope (SIF-H290S; Olympus, Tokyo, Japan) to facilitate stone removal. Carbon dioxide enterography alone failed to clearly delineate the route to the blind end owing to overlapping intestinal gas shadows. Therefore, DSI was also performed, enhancing the visualization of the anastomotic branch and enabling clear identification of the correct route ( Fig. 2 , Video 1 ).

**Fig. 1:**
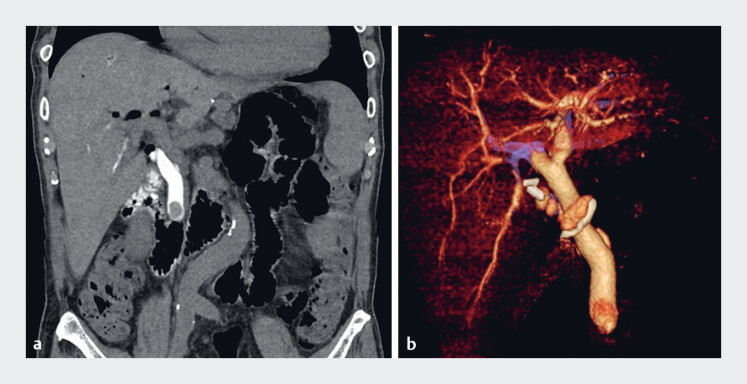
Imaging findings of a patient with common bile duct stones. **a** Coronal section of the drip infusion cholangiography-computed tomography showing a round filling defect in the common bile duct, consistent with a bile duct stone. **b** Three-dimensional reconstruction of the bile duct, providing detailed visualization of the stone's location and surrounding anatomy.

**Fig. 2:**
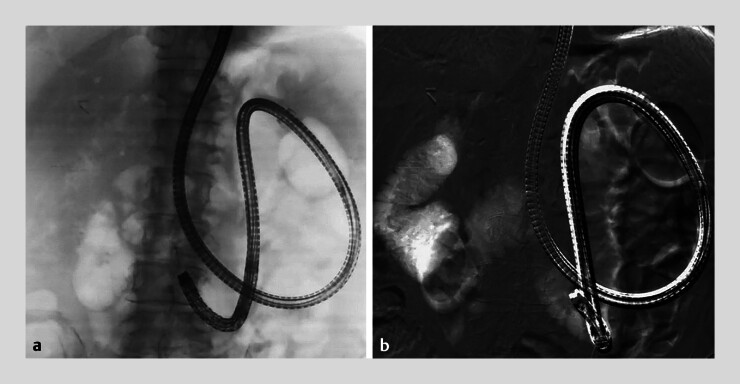
Comparison of carbon dioxide enterography and carbon dioxide digital subtraction enterography. **a** The route is obscured by overlapping intestinal gas in carbon dioxide enterography. **b** In carbon dioxide digital subtraction enterography, subtraction of the vertebral bodies and intestinal gas enhances route visualization, providing a clear view.

This video demonstrates the application of carbon dioxide enterography combined with digital subtraction imaging for route identification in a patient with post-Roux-en-Y reconstruction. The anastomotic branch was initially unclear because of the overlapping intestinal gas shadows. After digital subtraction imaging was applied, the route to the blind end became clearly visible, facilitating precise navigation and successful common bile duct stone extraction.Video 1

Carbon dioxide enterography is a valuable tool for route selection during ERC. However, its efficacy may be limited in cases of excessive intestinal gas. Combining DSI and carbon dioxide enterography enhanced route visibility. By eliminating background noise generated by overlapping gas shadows, this combination can improve route selection and procedural outcomes.

Endoscopy_UCTN_Code_TTT_1AP_2AD

## Notes

Endoscopy E-Videos https://eref.thieme.de/e-videos
*E-Videos* is an open access online section of the journal *Endoscopy* , reporting on interesting cases and new techniques in gastroenterological endoscopy. All papers include a high-quality video and are published with a Creative Commons CC-BY license. Endoscopy E-Videos qualify for HINARI discounts and waivers and eligibility is automatically checked during the submission process. We grant 100% waivers to articles whose corresponding authors are based in Group A countries and 50% waivers to those who are based in Group B countries as classified by Research4Life (see: https://www.research4life.org/access/eligibility/ ). This section has its own submission website at https://mc.manuscriptcentral.com/e-videos .
